# Novel compound heterozygous mutations in the *MYO15A* gene in autosomal recessive hearing loss identified by whole-exome sequencing

**DOI:** 10.1186/1479-5876-11-284

**Published:** 2013-11-09

**Authors:** Xue Gao, Qing-yan Zhu, Yue-Shuai Song, Guo-Jian Wang, Yong-Yi Yuan, Feng Xin, Sha-Sha Huang, Dong-Yang Kang, Ming-Yu Han, Li-ping Guan, Jian-guo Zhang, Pu Dai

**Affiliations:** 1 Department of Otorhinolaryngology, Head and Neck Surgery, PLA General Hospital, 28# Fuxing Road, Beijing 100853, P. R. China; 2 Department of Otolaryngology, Hainan Branch of PLA General Hospital, Sanya 572000, P. R. China; 3 Department of Otorhinolaryngology, the Second Artillery General Hospital, 16# Xin Wai Da Jie, Beijing 100088, P. R. China; 4 BGI–Shenzhen, Shenzhen 518083, China; 5 T-Life Research Center, Fudan University, Shanghai 200433, China

**Keywords:** Autosomal recessive sensorineural hearing loss, Whole-exome sequencing, *MYO15A*

## Abstract

**Background:**

Inherited genetic defects play an important role in congenital hearing loss, contributing to about 60% of deafness occurring in infants. Hereditary nonsyndromic hearing loss is highly heterogeneous, and most patients with a presumed genetic etiology lack a specific molecular diagnosis.

**Methods:**

By whole exome sequencing, we identified responsible gene of family 4794 with autosomal recessively nonsyndromic hearing loss (ARNSHL). We also used DNA from 56 Chinese familial patients with ARNSHL (autosomal recessive nonsyndromic hearing loss) and 108 ethnicity-matched negative samples to perform extended variants analysis.

**Results:**

We identified *MYO15A* c.IVS25 + 3G > A and c.8375 T > C (p.V2792A) as the disease-causing mutations. Both mutations co-segregated with hearing loss in family 4794, but were absent in the 56 index patients and 108 ethnicity-matched controls.

**Conclusions:**

Our results demonstrated that the hearing loss of family 4794 was caused by novel compound heterozygous mutations in *MYO15A*.

## Introduction

Hearing loss is a common sensory defect that can significantly impact quality of life. The majority of congenital cases are attributable to genetic factors [1]. Nonsyndromic hereditary forms, in which the hearing loss is the only clinical sign, are genetically heterogeneous. Autosomal recessive nonsyndromic hearing loss (ARNSHL) is the most common type and accounts for ~80% of cases of inherited hearing loss. To date, 61 genes and more than 100 genetic loci have been implicated in ARNSHL (see URL1). For many decades, linkage analysis has been the most powerful and widely used strategy to identify the gene defects responsible for inherited disorders. However, this approach is time consuming and requires the availability of cohorts of homogeneous and informative, possibly large families. As the molecular basis of deafness in most of the Chinese pedigrees is unsolved, we predict that many new deafness genes and mutations remain to be identified.

Whole-exome sequencing (WES) has become a highly efficient strategy for identifying novel causative genes and mutations involved in heritable disease [2]. Both simple nonsyndromic and complex syndromic forms of hearing loss can be resolved efficiently using WES, especially in small families with distinct and interesting phenotypes that were once too small to map [3]. To date, nine syndromic or nonsyndromic deafness genes have been identified using targeted genomic enrichment and next-generation sequencing: *TPRN*, *GPSM2*, *CEACAM16*, *SMPX*, *HSD17B4*, *HARS2*, *MASP1*, *DNMT1*, and *TSPEAR*[4-12]. As WES typically identifies many thousands of exomic variants, a selection strategy is important to facilitate the identification of the mutation that causes the disease [13,14]. With ARSNHL, WES of several affected and healthy members from one family may be powerful for quickly identifying new susceptibility genes or mutations.

*MYO15A* has 66 exons and its coding protein, myosin XVa, is composed of 3,530 amino acids, and is critical for the formation of stereocilia in hair cells of the cochlea. In the organ of Corti, myosin XVa is localized exclusively at the tips of stereocilia and is a motor protein that uses energy from ATP hydrolysis to move along actin filaments [15]. The tip of a stereocilium is the site of stereocilia growth and one of the proposed sites of mechano-electrical transduction [15]. Myosin XVa-deficient mice were reported to lack any links between stereocilia, suggesting a complete disruption of the mechanotransduction machinery [16]. Therefore, it was assumed that myosin XVa is required for proper formation and function of the mechanotransduction apparatus. Mutations in this gene cause DFNB3 hearing loss in individuals from different populations worldwide [17].

Here, we report a family with two siblings affected by severe-to-profound sensorineural hearing loss. Mutations in *GJB2* and *SLC26A4* were previously excluded. As the family is small and non-consanguineous, neither linkage analysis nor homozygosity mapping would have been informative for identifying the causative gene. Therefore, we used WES to identify the gene responsible for deafness in this family. WES was carried out in four members of family 4794 (two affected siblings and their parents), followed by validation. The results identified two novel compound heterozygous disease-segregating mutations, c.IVS25 + 3G > A and c.8375 T > C (p.V2792A), in the *MYO15A* gene. To exclude the possibility that these mutations were polymorphisms, DNA samples of 56 affected and 108 unaffected individuals were also analyzed. This is the first study to identify *MYO15A* mutations as the ARNSHL-associated gene in the Chinese deaf population.

## Methods and materials

### Clinical data

Family 4794 is a two-generation Chinese family with autosomal recessive prelingual non-syndromic sensorineural hearing loss. To screen for candidate mutations, we used 108 ethnicity-matched controls and 56 affected DNA samples from the Department of Otolaryngology, Head and Neck Surgery, Chinese PLA General Hospital. The 56 index patients were from families presenting with ARNSHL and in whom mutations of *GJB2* and *SLC26A4* had been previously excluded. Fully informed written consent was obtained from each subject. The study was approved by the Chinese PLA General Hospital Research Ethics Committee. Medical histories of the members of family 4794 were obtained using a questionnaire regarding the following aspects of the condition Otoscopy, physical examination, and pure tone audiometric examination (at frequencies from 250 to 8000 Hz) were performed to identify the phenotype. Immittance testing was applied to evaluate middle-ear pressure, ear canal volumes, and tympanic membrane mobility. Physical examination of all members revealed no signs of systemic illness or dysmorphic features. Computed tomography (CT) scan of the temporal bone was performed. The diagnosis of profound sensorineural hearing impairment was made according to the ICD-10 criteria based on audiometric examination. Tandem gait and Romberg tests were performed to evaluate balance.

### Preparation of DNA

All genomic DNA was extracted from peripheral blood using a blood DNA extraction kit according to the protocol provided by the manufacturer (TianGen, Beijing, China).

### Whole-exome capture and sequencing

Whole-exome capture and sequencing have been described in detail previously [18]. In brief, genomic DNA was captured using the NimblegenSeqCap EZ Library (64 Mb for I1, I2, II:1, II:2). DNA was sheared, ligated to adaptors, extracted, amplified by ligation-mediated PCR (LM-PCR), and then hybridized to the Nimblegen SeqCap EZ Library for enrichment. The each captured library was loaded onto the Illumina Hiseq2000 platform. Raw image files were processed by Illumina base calling software 1.7.

Read mapping, variant detection, filtering, and annotation have been described in detail previously [18]. Under the assumption of an autosomal-recessive pattern of inheritance, only variants that were homozygous or compound heterozygous in the two affected siblings and heterozygous in their parents were selected as candidates.

### Mutation validation

After filtering against multiple databases, Sanger sequencing was used to determine if any of the potential novel mutations in known causative genes of ARNSHL co-segregated with the disease phenotype in this family. Direct polymerase chain reaction (PCR) products were sequenced using Bigdye terminator v3.1 cycle sequencing kits (Applied Biosystems, Foster City, CA) and analyzed using an ABI 3700XL Genetic Analyzer.

### Mutational analysis

Segregation of the mutations was studied in all family members. In addition, 108 negative samples and 56 ARNSHL families were also screened for the mutations by direct sequencing. Genotyping for c.IVS25 + 3G > A and c.8375 T > C was performed by PCR (Additional file 1: Table S1) and detected by bidirectional sequencing of the amplified fragments using an automated DNA sequencer (ABI 3100; Applied Biosystems). Nucleotide alteration(s) were identified by sequence alignment with the *MYO15A* GenBank sequence (NM_016239) using Genetool software.

### Multiple sequence alignment

Multiple sequence alignment was performed according to a Homologene program with default settings and the sequences NP_057323.3 (*H. sapiens*), XP_0002888352.1 (*M. mulatta*), XP_003315632.1 (*P. troglodytes*), XP_002707808.1 (*B. taurus*), XP_536660.3 (*C. lupus*), NP_034992.2 (*M. musculus*), XP_577100.2 (*R. norvegicus*), XP_414818.3 (*G. gallus*), and XP_001924051 (*D. rerio*).

(http://www.ncbi.nlm.nih.gov/homologene?cmd=Retrieve&dopt=MultipleAlignment&list_uids=56504)

### Splice site prediction

We used Fruitfly (see URL2) analysis to evaluate the effect of c.IVS25 + 3 G > A on splice site. A 2.0 kb genomic DNA fragment encompassing the region harboring the mutation was involved.

### Model building and structural-based analysis

Three-dimensional (3D) modeling of the human wild type and p.V2792A mutation were performed using SWISS-MODEL, an automated homology modeling program (see URL3). This study used the automatic modeling approach to apply the complete protein sequence of human myosin XVa, including its 3519 amino acids and its mutation, which are available in the NCBI GenBank (NP_057323.3) in FASTA format. Data obtained by the homology models were visualized using Swiss-PdbViewer 4.1.

## Results

### Clinical presentation of family 4794

**Figure 1:**
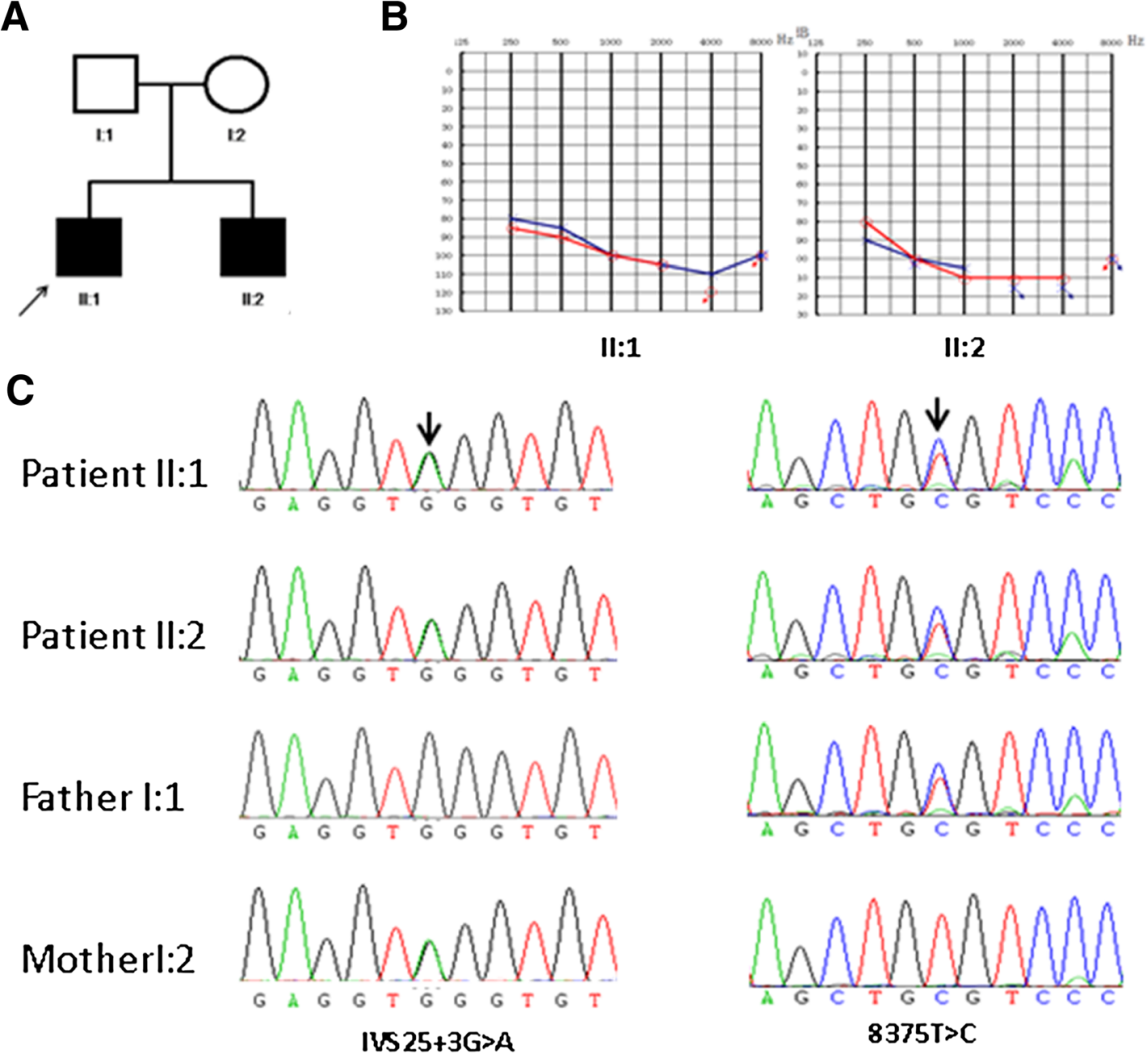
**Combined figure. A.** Pedigree of Family 4792 with ARNSHL Affected subjects are denoted in black. Arrow indicates the proband; **B.** Audiogram of affected subjects showed hearing loss ranged from severe to profound.; **C.** Electropherograms analysis of *MYO15A* in family 4792 showing the compound heterozygous mutations (c.IVS25 + 3G > A and c.8375C > T) co-segregated with the phenotype.

Family 4794 includes two affected siblings (31 and 25 years old) and two unaffected parents (Figure 1A). Audiograms of the affected siblings showed that the hearing loss was bilateral and severe to profound (Figure 1B). The affected individuals did not have delays in gross motor development; neither did they have balance problems. Tandem walking was normal, and the Romberg test was negative. CT scan of the temporal bone in the proband excluded inner-ear malformations. The parents reported no history of stillbirth or miscarriage. Physical examination of all family members revealed no signs of systemic illness or dysmorphic features. The remaining examination results were normal. This phenotype is consistent with that reported previously for DFNB3 [19]. No mutations were found in *SLC26A4* and *GJB2*, and we proceeded to sequence the whole exomes of the two patients.

### Whole-exome sequencing

We applied whole-exome sequencing to the two affected siblings and two unaffected parents: I1, I2, II:1, and II:2. The reads were aligned with the human genome reference sequence. On average, we generated 8.71 billion bases of sequence with mean target region depths of 74.5. Approximately 99.2% (4805 mb in length) of the targeted bases were covered sufficiently to pass our thresholds for calling SNPs and short insertions or deletions (indels). After identification of variants, we focused only on non-synonymous variants, splice acceptor and donor site mutations, and frameshift coding indels, which were more likely to be pathogenic than others, especially those in homozygous or compound heterozygous mode (Additional file 2: Table S2).

On average, we identified 150,306 SNPs in the coding regions and 3,259 variants in introns that may affect splicing (within 10 bp of the intron/exon junction) for each sample (Additional file 3: Table S3). Then we compared these variants with the dbSNP135 (see URL4), HapMap project (see URL5), 1000 Genome Project (see URL6), and YH database (see URL7). We identified potential candidate variants by selecting the homozygous or compound heterozygous variants found in both affected siblings while unaffected parents contributed equally. Under the autosomal recessive mode of inheritance, we identified 67 genes with compound heterozygous variants and 16 genes with homozygous variants. Then we compared these variants to reported nonsyndromic hereditary hearing loss genes; 2 mutations (c.IVS25 + 3G > A, c.8375 T > C) were found in a previously reported deafness-related gene, *MYO15A*.

### Mutation detection and analysis

According to the Berkeley Drosophila Genome Project (Fruitfly), a change in the splice donor sequence from GG to AG in intron 25 (c.IVS25 + 3 G > A) of *MYO15A*, detected in the present family, is predicted to result in an increase in splice site recognition by the splicing factor from 0.58 to 0.96, probably influencing the formation of the splice donor site.

**Figure 2:**
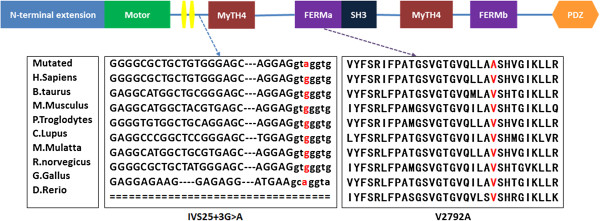
**Schematic structure of Myosin XVa and conservation analysis.** Diagram of the human MYO15A domains which consists of an alternatively spliced 1223-amino-acid N-terminus encoded by exon 2, a motor domain, two IQ motifs, and two MyTH4 domains, two FERM domains, a PDZ domain and an SH3 domain; IVS25 + 3G > A occur between IQ and MyTH4 domain, V2792A occur in the FERMa domain. Protein or sequence alignment showed conservation of residues MYO15A IVS25 + 3 and V2792 across nine species. These two mutations occur at an evolutionarily conserved nucleotide or amino acid (in red).

The c.8375 T > C substitution occurs in exon 47 of *MYO15A* and results in a single amino acid substitution, valine to alanine (p.V2792A), within the FERMa domain. The V2792 amino acid is conserved across mammalian species, including human, chimpanzee, dog, mouse, and rat, as depicted in Figure 2. c.8375 T > C (p.V2792A) was predicted to be damaging by SIFT and Polyphen2 (see URL8,9).

Both mutations affect the highly preserved residues (Figure 2). Sanger sequencing validated the two affected siblings’ mutations and demonstrated that the two parents were heterozygous carriers of c.IVS25 + 3G > A (mother) and c.8375 T > C (father), showing complete co-segregation of the mutation with the phenotype (Figure 1C). The two mutations were absent both in 108 negative samples and in 56 ARNSHL families. These observations suggest that the mutation V2792A is likely to have a detrimental effect on the protein.

### Structure modeling of p.V2792

**Figure 3:**
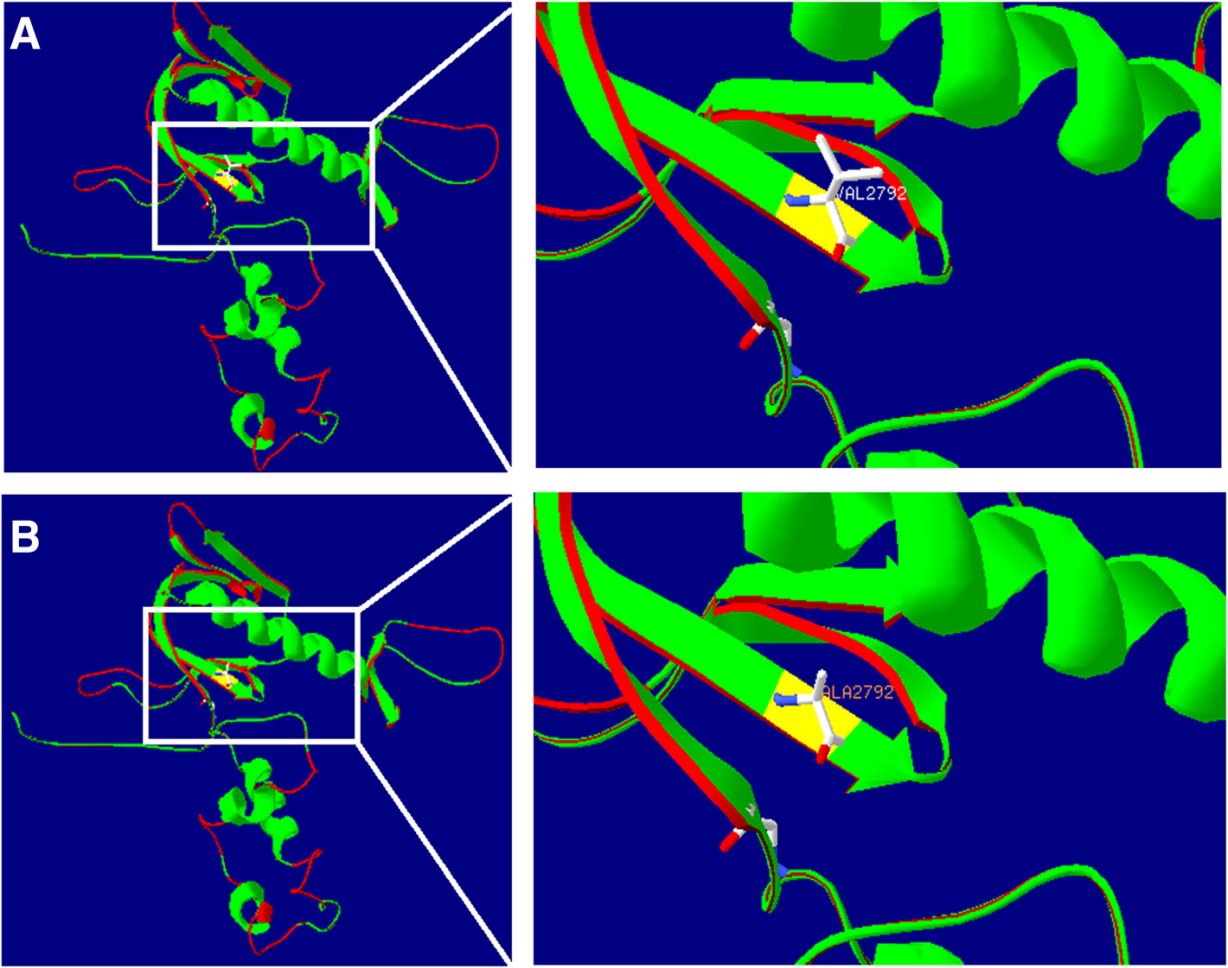
**Structure of wild**-**type and mutant 2792 of Myosin XVa. A:** wild-type V2792 has side chain; **B:** mutant A2792 has no side chain (Created by SWISS-MODEL and shown with Swiss-PdbViewer 4.1).

A molecular model of myosin XVa was constructed based on the crystal structure of the talin head FERM domain (PDB ID:3ivf), a protein domain that binds F-actin in vitro and in vivo at a specific site within the actin filament. The constructed model covered the target sequence of myosin XVa (residues 2685–2896). Although the sequence identity between the target and the template was 14.16%, lower than the average 25%, the target area’s sequence similarity is high. Using the Swiss-Pdb Viewer 4.1, we show that the mutation is predicted to perturb the amino acid side chain because of the substitution of valine to alanine. This region of the protein is predicted to be highly hydrophobic, as previously shown in a hydrophobicity plot (Figure 3).

These data, together with the clinical presentation of the two affected siblings, clearly indicate that *MYO15A* mutations are the cause of the ARNSHL in this family.

## Discussion

Despite previous intensive linkage analysis and candidate gene screening, a large proportion of ARNSHL remain genetically unexplained. WES provides unprecedented opportunities to identify causative DNA mutations in rare heritable disorders. This report describes a Chinese family whose hearing impairment is caused by novel compound heterozygous mutations in *MYO15A* gene. Mutations in this gene have previously been described to cause autosomal recessive hearing loss.

Human myosin XVa consists of an N-terminal domain (amino acids 1–1,223), motor domain, neck region, and tail domain. The motor domain (amino acids 1,224–1,899) contains two binding sites for ATP and actin, the switches I and II helix, a relay helix, an SH3 helix, an SH1/SH2 helix, and a converter domain. The neck region contains two light-chain binding motifs (1,909–1,942). The tail region contains two MyTH4 (myosin tail homology 4) domains (2,066–2,174 and 3,051–3,161), two FERM domains (2,687–2,867 and 3,217–3,497) that have a role in binding of cytoskeletal proteins to cytoplasmic domains of transmembrane proteins, a putative SH3 domain (2,865–2,959), and a C-terminal class I PDZ-binding motif [20,21]. The myosin XVa protein has been shown to be integral for development and elongation of the stereocilia through delivery of whirlin to the tips of the stereocilia. Whirlin binds to the SH3-MYTH4-FERM-domain-containing region of the myosin XVa protein and regulates actin filament elongation. There have been several reports of mutations in *MYO15A* causing hearing loss [17,21-27]. Forty-seven mutations have previously been reported in *MYO15A*, and primarily occur in the motor domain.

The c.8375 T > C (p.V2792A) mutation is located within exon 47 and predicted to lie in the first FERM domain of myosin XVa. Three mutations in this domain (Q2716H, D2720H, D2823N) have been reported. The FERM domain is a widespread protein module involved in localizing protein to the plasma membrane, and its amino acid sequence is highly conserved [28].

In this family, a novel splice site mutation c.IVS25 + 3G > A was found in intron 25 of *MYO15A* and is predicted to change the highly conserved donor splice site of intron 25 from GG to AG. Mutations at splice sites are commonly associated with diminished amounts or abnormal maturation of mRNA, resulting in either exon skipping or cryptic splice site activation [29]. These possibilities have not been investigated, and RNA studies are not possible in this instance because we cannot obtain fresh peripheral blood samples.

In summary, we have reported the clinical and genetic characteristics of a non-consanguineous Chinese family with ARNSHL by WES. To identify pathogenic variants, we consecutively filtered these variants by subjecting them to an analytical pipeline for high-confidence variant calling and annotation and identified novel compound heterozygous mutations in *MYO15A*. The identification of additional novel mutations in *MYO15A* further confirms the crucial role of *MYO15A* in auditory function. These results support sequence analysis of *MYO15A* in the clinical diagnostic testing of individuals with ARNSHL. Pre-implantation genetic diagnosis might be available for these families.

## Web resources

The URLs presented are as follows:

1. Hereditary Hearing Loss, http://hereditaryhearingloss.org/

2. Fruitfly, http://www.fruitfly.org/seq_tools/splice.html

3. SWISS-MODEL, http://swissmodel.expasy.org/workspace/

4. dbSNP135, http://www.ncbi.nlm.nih.gov/projects/SNP/

5. HapMap project, ftp://ftp.ncbi.nlm.nih.gov/hapmap

6. 1000 Genome Project, ftp://ftp.1000genomes.ebi.ac.uk/vol1/ftp

7. YH database, http://yh.genomics.org.cn/

8. SIFT, http://sift.bii.a-star.edu.sg/

9. Polyphen2, http://genetics.bwh.harvard.edu/pph2/

## Abbreviations

ARNSHL: Autosomal recessive nonsyndromic hearing loss; NGS: Next-generation sequencing; WES: Next-generation sequencing; CT: Computed tomography; PCR: Polymerase chain reaction.

## Competing interests

The authors declare that they have no competing interests.

## Authors’ contributions

Conceived and designed the experiments: PD XG. Performed the experiments: XG QYZ YSS GJW FX DYK. Analyzed the data: XG YYY SSH MYH LPG. Contributed reagents/materials/analysis tools: JGZ. Wrote the paper: XG PD. All authors read and approved the final manuscript.

## Authors’ information

XueGao, Qing-yan Zhu and Yue-Shuai Song are listed as co-first authors.

## Supplementary Material

Additional file 1: Table S1 Primers used for potential mutations amplification. Click here for file

Additional file 2: Table S2 Overview of data production using WES. Click here for file

Additional file 3: Table S3 Summary of SNPs for 4 exome capture samples. Click here for file
