# Supplementary material for: Novel compound heterozygous mutations in the MYO15A gene in autosomal recessive hearing loss identified by whole-exome sequencing
Source: J Transl Med. 2013 Nov 9;11:284. doi: 10.1186/1479-5876-11-284 (PMC3828584; doi:10.1186/1479-5876-11-284)
Supplement: Additional file 1: Table S1 — Primers used for potential mutations amplification. [file 1479-5876-11-284-S1.docx]

**Supplemental Table 1 Primers used for potential mutations amplification**

|  | **Forward primer** | **Reverse primer** |
| --- | --- | --- |
| c.IVS25+3 G>A | TCCCAGAAATCCTCCTTGTG | TGTGGAAAGAGGAGGGAATG |
| c.8375T>C | GAACCAGCTGGACACACAGA | GCCTAGCTCAATCCCTTCCT |
