# Supplementary material for: Novel compound heterozygous mutations in the MYO15A gene in autosomal recessive hearing loss identified by whole-exome sequencing
Source: J Transl Med. 2013 Nov 9;11:284. doi: 10.1186/1479-5876-11-284 (PMC3828584; doi:10.1186/1479-5876-11-284)
Supplement: Additional file 2: Table S2 — Overview of data production using WES. [file 1479-5876-11-284-S2.docx]

**Supplemental Table 2 Overview of data production using WES**

| **Exome Capture Statistics** | **II:1** | **I:1** | **II:2** | **I:2** |
| --- | --- | --- | --- | --- |
| Target region (bp)^(1)^ | 64558893 | 64558893 | 64558893 | 64326610 |
| Raw reads | 103314922 | 103719856 | 90638686 | 89651828 |
| Raw data yield (Mb) | 9298 | 9335 | 8157 | 8069 |
| Reads mapped to genome | 91418687 | 96742848 | 82142672 | 83835308 |
| Reads mapped to target region^(2)^ | 69879028 | 72747967 | 62080488 | 62409099 |
| Data mapped to target region (Mb) | 5036.98 | 5228.20 | 4474.23 | 4479.83 |
| Mean depth of target region(X) | 78.02 | 80.98 | 69.30 | 69.64 |
| Coverage of target region (%) | 99.22 | 99.23 | 99.20 | 99.14 |
| Average read length (bp) | 89.97 | 89.92 | 89.97 | 89.93 |
| Rate of nucleotide mismatch (%) | 0.23 | 0.24 | 0.24 | 0.24 |
| Fraction of target covered >=4X (%) | 98.32 | 98.45 | 98.23 | 98.24 |
| Fraction of target covered >=10X (%) | 97.09 | 97.37 | 96.87 | 97.00 |
| Fraction of target covered >=20X (%) | 94.70 | 95.35 | 93.66 | 94.13 |
| Capture specificity (%)^(3)^ | 77.28 | 76.03 | 76.40 | 75.12 |
| Reads mapped to flanking region^(4)^ | 13120184 | 14758977 | 11713419 | 13150858 |
| Mean depth of flanking region(X) | 27.20 | 29.45 | 24.23 | 25.86 |
| Coverage of flanking region (%) | 97.85 | 98.14 | 97.63 | 97.98 |
| Fraction of flanking covered >=4X (%) | 90.19 | 92.64 | 88.56 | 91.73 |
| Fraction of flanking covered >=10X (%) | 69.55 | 74.31 | 65.83 | 71.50 |
| Fraction of flanking covered >=20X (%) | 44.80 | 49.04 | 40.30 | 44.56 |
| Fraction of unique mapped bases on or near target (%) | 90.34 | 89.88 | 89.34 | 89.33 |
| Duplication rate (%)^(5)^ | 9.63 | 4.38 | 7.27 | 4.07 |
| Mean depth of chrX(X) | 57.66 | 59.78 | 51.03 | 97.40 |
| Mean depth of chrY(X) | 205.52 | 188.14 | 181.09 | - |
| GC rate (%) | 46.27 | 46.88 | 46.28 | 46.12 |
| Gender test result | M | M | M | F |
