# Supplementary material for: Novel compound heterozygous mutations in the MYO15A gene in autosomal recessive hearing loss identified by whole-exome sequencing
Source: J Transl Med. 2013 Nov 9;11:284. doi: 10.1186/1479-5876-11-284 (PMC3828584; doi:10.1186/1479-5876-11-284)
Supplement: Additional file 3: Table S3 — Summary of SNPs for 4 exome capture samples. [file 1479-5876-11-284-S3.docx]

**Supplemental Table 3 Summary of SNPs for 4 exome capture samples**

| **Categories** | **II:1** | **I:1** | **II:2** | **I:2** |
| --- | --- | --- | --- | --- |
| Number of genomic positions for calling SNPs^(1)^ | 166510055 | 166510055 | 166510055 | 165853891 |
| Number of high-confidence genotypes^(2)^ | 155787761 | 155041628 | 156979317 | 156305612 |
| Number of high-confidence genotypes in TR | 63066652 | 63036392 | 63197142 | 62972217 |
| Total number of SNPs | 150277 | 146217 | 154042 | 150686 |
| Nonsense | 173 | 166 | 162 | 172 |
| Readthrough | 49 | 54 | 51 | 58 |
| Missense | 13155 | 12926 | 12943 | 12897 |
| Splice site^(3)^ | 3240 | 3278 | 3292 | 3224 |
| 5-UTR | 14645 | 14316 | 15070 | 14660 |
| 3-UTR | 19186 | 18795 | 19354 | 19154 |
| Synonymous-coding | 6520 | 6372 | 6378 | 6390 |
| Intron | 85006 | 82241 | 88219 | 85801 |
| Intergenic | 8303 | 8069 | 8573 | 8330 |
| Hom | 57964 | 56187 | 59392 | 57579 |
| Het | 92313 | 90030 | 94650 | 93107 |
